# Fertility-Preserving Surgery in Patients with Early Stage Cervical Carcinoma

**DOI:** 10.5402/2012/817065

**Published:** 2012-12-18

**Authors:** Spyridon Kardakis

**Affiliations:** Department of Obstetrics and Gynaecology, Oncologic Clinic, Västerås Hospital, 72212 Västerås, Sweden

## Abstract

Fertility preservation is an important issue for patients in reproductive age with early stage cervical cancer. In view of recent developments, our purpose was to review and discuss available surgical alternatives. A literature search was conducted using PUBMED, including papers between 1980 and December 2011. In patients with stage IA1 cervical cancer, conization is a valid alternative. Patients with stage IA2-IB1 disease can be conservatively treated by radical trachelectomy. This is as well-established conservative approach and appears to be safe and effective in allowing a high chance of conception. Prematurity is the most serious issue in pregnancies following trachelectomy. Less invasive options such as simple trachelectomy or conization seem to be feasible for stages IA2-IB1, but more and better evidence is needed. Neoadjuvant therapy might allow conservative surgery to be performed also in patients with more extensive lesions. Ovarian transposition is important when adjuvant radiation is needed. In conclusion, available literature shows that there are interesting fertility-sparing treatment alternatives to the “golden standard” for the management of early cervical cancer in young women.

## 1. Introduction

Cervical cancer is the seventh most common malignancy in developed countries, and the second most common cancer in developing countries [1]. In 2004, 30,570 new cases of invasive cervical cancer were diagnosed in the European Union [1]. In 2012, the estimated new cases in the USA are 12,170, and the estimated deaths 4,220 [2]. Higher incidence occurs in countries where an effective screening program is not present [1]. In the USA, the incidence of cervical cancer is about 6.8 per 100,000 person and the mortality 2.4/100,000. Gynecological malignancies often affect women in reproductive age and about 28% of all cervical cancers is diagnosed prior to 40 years of age [3]. Where a screening program is present, the disease is often diagnosed in early stages with high survival rates. In the USA, between 2001 and 2007 the 5-year survival for localized disease in white women under 50 years old was 94.2% [4]. The golden standard treatment of early stage disease ranges from simple hysterectomy (stage IA1) to a radical hysterectomy (RH) and pelvic lymphadenectomy (IA2 to IB1). Nevertheless, the high survival rates and the delayed childbearing in our society result in more cervical cancer patients who desire preserving their fertility. Luckily, fertility sparing treatment approaches are available for a large part of cases. Cervical conization is a feasible treatment for stage IA1 and radical trachelectomy with laparoscopic lymphadenectomy has become a surgical alternative for stages IA2 and IB1. The aim of this paper was to review available literature on fertility preserving surgery in early cervical cancer, focusing on safety and reproductive outcomes.

## 2. Stage IA1: Conization

Nodal and parametrial tissue involvement is rare in very early stage disease (stage IA1) and the standard treatment is a simple hysterectomy. Conization has been suggested as a conservative surgical alternative and fertility sparing approach. Candidates for conization are patients with stage IA1 cervical cancer without lymphovascular space involvement at the pathological examination, negative margins, and normal endocervical curettage. Although lymphovascular space invasion does not affect staging, its presence increases the risk of lymph node metastasis and the standard treatment has been RH and pelvic node dissection. Radical trachelectomy with pelvic lymph node dissection is the treatment of choice when patient desires to preserve fertility.

Several authors have reported absence of node metastasis when stromal invasion is less than 4 mm [5–7]. These lesions have less than 1% incidence of lymph nodal metastasis, and, within this group, lymph vascular invasion increases the risk.

The lymph vascular space involvement (LVSI) in patients with early stage disease is associated with pelvic nodal metastasis and the quantity of LVSI, as defined by the percentage of all cervical histopathologic sections containing LVSI, correlates significantly with the risk of nodal metastases [8]. If lymph vascular invasion is present radical trachelectomy with lymph node dissection should be considered.

Conization is controversial in cases of adenocarcinoma because of the difficulty of establishing a pathologic diagnosis of microinvasion from a glandular lesion. Reports suggest that conization may be performed on patients with both squamous carcinoma and adenocarcinoma [9]. Bisseling et al. [8] report no recurrence at 72 months follow-up in 16 patients with stage IA1 adenocarcinoma who underwent conization alone. The same authors recommend conization and pelvic node dissection where lymph vascular space involvement is present. 

The efficacy of laser conization, loop electrosurgical excision procedure (LEEP), and cold-knife conization is similar [10]. Mathevet et al. demonstrated that there was no major difference in obstetrical outcome among the three techniques [11]. However, it is known that frequently a diathermy loop excision can produce a thermal effect at the margin, making evaluation of the margins difficult.

Conization appears safe in IA1 stage, as no difference in survival rates between this approach and hysterectomy has been reported in many studies. Small series have shown a risk of recurrent invasive cancer below 0.5%, and a risk of recurrence of preinvasive disease between 7 and 10% [12, 13]. Tseng et al. [7] reported 12 cases of stage IA1 cervical cancer treated with conization with an overall survival rate of 100% at 6.7 years. Pahisa et al. [14] report 4 conizations in patients with adenocarcinoma stage IA1. At 16–72 months of follow-up, one case of microinvasive recurrence was reported, occurring 5 years after conization. Wright et al. [15] confirm these excellent results in a recent study of 1,409 patients. In this study the survival rate after a five-year follow-up was 99% (95% CI 96–99%) following hysterectomy and 98% (95% CI 97–99%) following conization. Recurrence rate was 3%.

 Conization is associated with an increased risk of preterm delivery but no major differences in obstetrical outcome among different techniques of conization are demonstrated [11]. Two meta-analyses [16, 17] evaluate the obstetric outcome after cervical surgery. Both studies reported an increased risk of preterm delivery (relative risk [RR], 2.59; 95% CI, 1.8–3.72 [17]; RR, 2.78; 95% CI, 1.72–4.51 [17]) and low birth weight (RR, 2.53; 95% CI, 1.19–5.36 [16]; RR, 2.86; 95% CI, 1.37–5.97 [17]) in patients with a history of knife conization.

## 3. Stage IA2-IB1: Trachelectomy

Patients with cervical cancer at stages IA2 and IB1 have a high incidence of pelvic lymph node metastases and pelvic node dissection is necessary. The conventional treatment in these stages consists of RH and bilateral pelvic lymph node dissection. Vaginal radical trachelectomy (VRT) with pelvic lymphadenectomy, as a fertility sparing treatment of early-stage cervical carcinoma, was first described in 1987 by Dargent [18]. Sonoda et al. refer that 40% of patients who underwent a RH at their institution would have been candidates for a VRT [19]. 

During surgery spread to the lymph nodes should always be assessed therefore a laparoscopic pelvic lymph node dissection is performed before trachelectomy. The lymph nodes from the external and internal iliac and obturator region are removed and the presence of metastasis is evaluated by a frozen section. If lymph nodes are negative, the trachelectomy is performed. In case of positive lymph nodes an RH followed by chemo-radiotherapy or definitive chemo-radiotherapy is the treatment of choice.

Different surgical approaches are possible. Trachelectomy can be performed vaginally or abdominally with open or laparoscopic technique and it is usually accompanied by cervical cerclage placement. Because of the limitations such as cervical stenosis, crypto-menorrhea and dysmenorrheal, cerclage is also recommended in second trimester of pregnancy. Lymphadenectomy is performed either laparoscopically or by laparotomy. The assessment of lymph nodes by detection of sentinel lymph node has recently suggested. The sensitivity and specificity for the detection of lymph node metastases for sentinel node biopsy is 91% and 100%, respectively.

### 3.1. Indications and Patient Selection Criteria

Trachelectomy has been adopted by many oncological centers all over the world with good oncological and obstetrical results. The selection of patients by adequate preoperative evaluation is an important process before a decision regarding conservative treatment is taken. The extension of the lesion is of great importance; the tumor should be small in size and confined to the cervix without parametrial invasion or spread to the uterine corpus. A recurrence rate of 19% has been reported for patients with lesions >2 cm and 25% for those with lesions >2 cm and depth of invasion >1 cm [20, 21].

It is controversial whether imaging studies are more useful than clinical examination to assess tumor size and local spread. Colposcopy can also be performed and it is useful in assessing the exocervical diameter and the extension to the vagina. Some data suggest that a preoperative assessment with magnetic resonance imaging (MRI) is the most adequate diagnostic option of measurement of lesion diameters, amount of cervical stroma infiltration, and parametrial invasion. A prospective study including 208 women who underwent MRI and CT prior to surgery [22] reports that MRI correlated more closely with surgical pathologic findings than CT or physical examination. All three modalities overestimated tumor size. MRI is also useful for the detection of the endocervical extension of the lesion in relation to the isthmus. Many centers suggest that infiltration of less than half of the stroma is a safe limit because it allows the free margin of 1 cm. One cm cervical stroma is necessary to reduce the risk for premature delivery [23–25]. In cases where the upper margin of the lesion is less than 1 cm from the isthmus, neoadjuvant chemotherapy can be offered before VRT. Vaginal and rectal ultrasound can also be used to assess the size of the tumor [26].

The preoperative evaluation of lymph nodes is commonly performed with CT, but PET and PET/CT are the most adequate imaging modalities and they have replaced MRI and lymphangiography. A meta-analysis of 72 studies including 5042 women with cervical cancer found that PET has a better sensitivity and specificity for the detection of lymph node metastases (sensitivity: 75%, specificity: 98%); than MRI (56% and 93%) or CT (58% and 92%) [27]. Integrated PET/CT may be more sensitive than PET alone for detection of nodal metastases, particularly for pelvic lymph nodes [28].

Several articles have suggested criteria that should be satisfied for considering a radical trachelectomy. It is important that patients have a strong desire to preserve fertility and have been adequately informed.

Radical trachelectomy is recommended for stage IA2 and IB1. When lymph vascular space involvement is present in stage IA1, conization is not an adequate treatment and radical trachelectomy should be performed.

The lesion should be less than 2 cm and confined to the cervix with stromal infiltration less than 10 mm. Absence of lymph nodal involvement is necessary. The procedure is not recommended for small cell carcinoma or sarcoma such as in cases with capillary space involvement. Although lymph vascular space involvement is a negative prognostic factor when present alone it is not an absolute contraindication [29] 

There is a general controversy whether adenocarcinoma histology is associated with worse prognosis than squamous cell carcinoma [29–32]. Adenocarcinoma often involves the endocervix and it can be difficult to eliminate the entire lesion. Although all series included cases of adenocarcinoma, none have addressed the influence of histology on outcome. Helpman et al. [33] in a recent large series, where the influence of histology on outcome is determinate, report no significant difference in recurrence-free survival between adenocarcinoma and squamous cell carcinoma.

### 3.2. Morbidity and Complications

The radical trachelectomy present a risk for intraoperative complications to ureter, bladder, and rectum. However the intraoperative complications are rare. Plante et al. [34] report a 5.6% of intraoperative complications in a large series which includes 125 patients underwent radical trachelectomy. In this study a total of 7 complications are described, four related to laparoscopy and only 3 to the trachelectomy, two bladder lesions and one uncontrollable bleeding. No significant differences between intraoperative and postoperative complications are reported between trachelectomy and RH [35]. Fewer blood transfusions, less total blood loss, and shortened hospital stays are associated with VRT [35]. 

Specific problems are associated with radical trachelectomy as dysmenorrhea, dysplastic smears, irregular bleeding, excessive vaginal discharge, problem with cerclage suture, isthmic stenosis, amenorrhea, and sexual inactivity.

### 3.3. Vaginal Radical Trachelectomy

Whether trachelectomy is effective and safe as RH is of great importance. The data in the literature suggests a low recurrence and death rate. A randomized study comparing this two surgical procedures is not feasible because it is difficult to have enough patients to reach statistical significance and because of ethical reasons. Alexander-Sefre et al. found no significant differences in 5-year overall survival rate and 5-year progression-free rate between radical trachelectomy and RH [36]. Lanowska et al. [37] report a 2.7% recurrence rate in a case series including 112 patients with early cervical cancer underwent VRT. Recurrence occurred in only 3 patients after 18 months follow-up. In the first patient for technical problems no parametrium could be removed on the right side. Carcinoma in situ was found in the endocervical margin of the trachelectomy specimen in the second patient but she did not accept hysterectomy and invasive carcinoma was found four months later. The third patient was diagnosed with cancer of 22 mm in diameter and insisted on VRT. 

We reviewed the case series regarding VRT as fertility sparing option (Tables 1 and 2). Trachelectomy has been offered in a total of 845 patients. In 40 cases, trachelectomy has been abandoned because of different reasons as metastatic nodes detected during lymphadenectomy or more extensive lesions and no adequate margins discovered at the time of surgery. Finally, 805 radical vaginal trachelectomies have been performed. The median follow-up was from 21 to 95 months in different studies with a total of 35 recurrences (4.3%) and 20 deaths (2.5%) reported.

### 3.4. Abdominal Radical Trachelectomy

Abdominal radical trachelectomy (ART) has a low intraoperative complication rate, making it possible to operate on cases with distorted cervicovaginal anatomy, providing larger parametrial resection. In addition it seems to be more familiar to gynecologic oncologists and it is easier to perform in nulliparous patients where advanced vaginal surgical skills are required to perform a vaginal operation. 

In 1997, Smith et al. [38] published the first two cases of ART. The largest series is reported by Li et al. [39], in 2011, and include 62 patients treated with ART. In this series there are no recurrence in 22.8 months of median follow-up. 

A total of 10 case series are published including 244 patients. The median follow-up is from 6 to 47 months. A total of 8 recurrences are reported with overall recurrence rate 3%. No deaths are reported (Tables 3 and 4).

### 3.5. Laparoscopic Radical Trachelectomy

Although open ART offers better resection of the margins, it is associated with longer hospitality stay, more blood loss, and wound complications compared with the VRT. Laparoscopic radical trachelectomy (LRT) is, in principle, identical to the ART and it has all the advantages of an abdominal approach combined to a mini invasive surgery. There are only a few small case series published; however the available data suggests that the laparoscopic way to perform a trachelectomy can be a safe alternative [40–46]. The largest series published by Kim et al. in 2010 [42] regarding 32 patients reports one recurrence and one death after a median follow-up of 31 months (Table 5).

### 3.6. Robotic Radical Trachelectomy

The dissection is extremely difficult and requires advanced surgical skills and expertise when performed with laparoscopic approach. The robotic approach offers better motion with finer instruments, precision, and three-dimensional image. All those factors are an advantage considering the complexity of this procedure. Furthermore contrary to the ART the uterine artery can be preserved and only the descending braches are transected maintaining the blood supply to the uterus. Recently some centers introduced the robotic approach and small case series are published. A total of six reports are published with overall 25 patients underwent robotic radical trachelectomy (RRT) (Table 6).

The first reported case series was published by Persson in 2008 [47], describing two patients with stage IB1 who underwent RRT without complications. Burnett et al. [48] report a series of 6 patients who operated with robotic laparoscopically assisted trachelectomy with preservation of fertility in 5 and one case of conversion in hysterectomy because of positive margins. Nick et al. [49] in a retrospective study compare the perioperative outcome between RRT and ART. In this study the robotic surgical approach resulted in less blood loss and decreased length of hospital stay with no compromise in histopathological outcome. 

The robotic approach seems to be safe but the number of patients is limited. The evaluable studies have not reported on obstetrical results because of a short follow-up time. The reproductive outcomes must be further evaluated.

#### 3.6.1. Infertility and Reproductive Outcome following Vaginal Radical Trachelectomy

Several large series report the obstetrical outcome after VRT (Table 7). An overall of 805 radical vaginal trachelectomies are performed. A total of 359 pregnancies in 217 patients have been reported, resulting in 229 births. At the moment of the studies 19 ongoing pregnancies are reported. 58 pregnancies have miscarried in the first trimester (17%) and this is comparable to the rate in the general population (16 to 20 percent). 24 patients (7%) had a second trimester miscarriage versus 4% of the general population. The overall miscarriage rate after trachelectomy is 24%.

A total of 229 live births are reported and 67% gave birth after 36 weeks. Premature delivery occurred in 23% versus 10% of the general population [50]. Only 8 of the 10 published reports report the exact number of pregnancies before 32 weeks. These include 150 live births with a 8.8% premature rate before 32 weeks. Between 32 and 36 weeks of gestation gave birth 23.3% of the women. 

The main problem is the prematurity in the obstetrical outcome. Second trimester miscarriage and premature rupture of membrane (PROM) with following premature labor are complications related to trachelectomy. The shortened cervix with absence of mucus facilitats the ascending infections and can result in chorioamnionitis and premature rupture of the membranes [51, 52]. Pregnancies after trachelectomy should be considered as high risk but no definitive guidelines regarding the management of these patients are published. Some authors suggest regular vaginal swabs every two weeks or the use of prophylactic antibiotics at 16 and 24 weeks, bed rest, and routine administration of steroids [53] Routine cytologies in asymptomatic women and sexual intercourse from the 20th week should be avoided because of the increase risk of infection. Follow-up with serial cervical ultrasound measurement has a predictive value of preterm labor and could be useful to decide the time for steroid administration. Cesarean section is recommended after 37 weeks. A vaginal delivery could be dangerous because of the possibility of a lateral cervical tear extending to the uterine arteries. Klemm et al. [54] demonstrated that the uterine perfusion after trachelectomy is unchanged and support no risk for intrauterine growth restriction. 

Infertility has been reported in 25–30% of patients after trachelectomy and possible causes include cervical cervical stenosis, decreased cervical mucus, and subclinical salpingitis [51, 55]. Cervical stenosis occur in 15% of patients who underwent trachelectomy and dilatation of the cervix can resolve the problem in the majority of the cases [55, 56]. Plante et al. [34] report infertility in 15 patients of 111 underwent trachelectomy but patients fertility was not proved before operation. Of those 40% were due to cervical factor, and the remaining 60% were unrelated to the trachelectomy.

Patients with infertility after trachelectomy can be pregnant with IUI or IVF but particular attention should be made to avoid multiple gestations considering the preexisting high risk of preterm delivery in this patients. Embryo transfers can be difficult in case of stenosis and specific care is required. A catheter can be placed in the cervix while the patient is being stimulated. Intraperitoneal insemination in the pouch of Douglas can be an option when tubes are patent and semen quality is sufficient. If it is impossible to perform transcervical embryo transfer with a severely stenotic cervical opening, a transmyometrial embryo transfer under ultrasound guidance can be performed. [57, 58] Gametes or zygotes intrafallopian transfer are also a therapeutic option.

### 3.7. Followup

Patient who underwent trachelectomy should been seen every 3 to 4 months for the first two years, then every 6 months. Some centers continue the follow-up every year after the first five years [59]. A follow-up with clinical and colposcopic examination and cervical cytology should be performed in every visit. Problematic can be also the interpretation of cytology. In about 58%–60% of smears atypical cells were found leading to false positive smear [59, 60]. Feratovic et al. [60] published the results of 223 cytology specimens. All cases reported as atypical glandular cells were endometrial stromal cells, tubal metaplasia, and lower uterine segment glandular cells. Some centers perform routine MRI at 6, 12, and 18 months. The interpretation of MRI findings can be difficult because of altered anatomy.

### 3.8. Postoperative Treatment and Ovarian Transposition

There are different prognostic factors for early stage cervical cancer and they are classified in intermediate- and high-risk factors for recurrent disease. If high risk factors (positive or close resection margins, positive lymph nodes, parametrial involvement) are identified an adjuvant radiation or chemo-radiotherapy is needed. Beiner and Covens [51] in a review report that approximately 10% of patients who underwent VRT would be candidates for adjuvant treatment. The included patients had either positive nodes, positive margins, or parametrial involvement on final pathology. Adjuvant therapy is associated with risk of premature ovarian failure.

Deep stromal invasion, large tumor size, and lymph vascular invasion are intermediate risk factors and if any of these are identified, adjuvant radiation decreases the risk of recurrence [61]. Whether adjuvant therapy can be avoided in some of these patients has not been determined. A conservative approach with close follow-up has a potential high risk of recurrence and the patient should be informed.

Ovarian transposition can be performed to avoid damage of ovarian tissue when radiation is needed after surgery. Laparoscopic lateral ovarian transposition is the simplest and most effective technique that can be used in patients with cervical cancer who will be undergoing pelvic radiation. Ovarian transposition is beneficial not only for preservation of fertility but also to prevent premature menopause. The preserving of ovarian function has successful rates about 90% after vaginal brachytherapy and 60% in patients undergoing pelvic radiation [62]. Ovarian failure rates reported after ovarian transposition were due to various factors, such as vascular compromise, type of radiation, external or brachytherapy, and dose [63]. Complications of ovarian transposition are chronic ovarian pain, ovarian cysts, and infraction of the fallopian tubes.

## 4. Stage IA2-IB1: Conization and Simple Trachelectomy

Patients with cervical cancer stage IA1 with LVSI, stage IA2, and IB1 are treated with radical trachelectomy and lymphadenectomy. Radical trachelectomy is associated with complications due to the removal of the parametrium which contains nerve fibers implicated in the innervations of bowel and bladder. Fertility outcomes are good but not as good as the results after conization. On the other hand, the utility of parametrial resection is controversial [84, 85]. Approximately 60% of patients undergoing trachelectomy have no residual disease in the final pathologic specimen after a diagnostic cone and less than 1% of patients with favorable pathologic characteristics have parametrial involvement [85].

Several retrospective studies report a low incidence of parametrial involvement (0 to 0.6%) in patients, with early stages of cervical cancer and favorable histopathological characteristics, who underwent RH [85–88]. In the largest study of 536 patients with tumor size <2 cm, depth invasion <10 mm, and negative pelvic lymph nodes, including any histology, only 0.6% had parametrial involvement [85]. Stegeman et al. [87] report 0% of parametrial involvement in 101 patients underwent RH. In this study patients with squamous, adenocarcinoma, adenosquamous, or clear cell histology, tumor size <2 cm, depth invasion <10 mm, no LVSI, and negative pelvic lymph nodes were included.

The resection of part of the parametrium is based on the presence of small lymph nodes in the tissue. Lanowska et al. [37], in a recent study, found an incidence of metastasis less than 1%. The presence of lymph node in the parametria was 7.1% and the authors found a statistically significant difference in the thickness of the parametrium between patients with and without lymph nodes. A radiological preoperative evaluation with measurement of parametrium volume using RMN could help for the selection of patients who could benefit of a less invasive surgery.

In the recent years some centers have adopted more conservative surgery using large cone or simply trachelectomy with a high successful pregnancy rate and encouraging oncologic results [89, 90]. There are only a few case reports about less radical fertility sparing surgery in early stages. In a recent small case series by Fagotti et al. [89], conization was performed in patients with cervical cancer stage IA2-IB1 and tumor <20 mm. A total of 17 patients were treated by excisional cone and laparoscopic lymphadenectomy. Lymph vascular space invasion was presented in 4 cases. No recurrences were observed after a median follow-up of 16 months (range 8–101 months). Two of five patients, attempting to conceive, had a spontaneous pregnancy and delivery. Rob et al. [90] report the results of 40 patients with early stage cervical cancer (stage IA1 to IB1) who underwent laparoscopic sentinel node identification followed by conization or trachelectomy. Negative sentinel lymph nodes had 34 patients (85%). A cone (stage IA1 with LVSI and stage IA2) or simple trachelectomy (stage IB1 less than 2 cm) was performed some days after definitive histopathologic confirmation of negative nodes. Only one recurrence has been reported after a mean follow-up of 47 months. The patient was in stage IB1 with LVSI. These recent studies report conization or simple trachelectomy and pelvic lymphadenectomy as a safe conservative approach but more studies are needed to confirm the safety of this method.

## 5. Conservative Surgery and Neoadjuvant Chemotherapy

Neoadjuvant chemotherapy before conservative surgery in early cervical cancer can reduce the tumor size prior trachelectomy or conization. Neoadjuvant chemotherapy in patients who did not meet the favorable pathological criteria has been reported by small series suggesting that this approach may be an option. Rob et al. [24] report 9 patients who underwent three cycles with isofosfamide and cisplatin or cisplatin and adriamycin. Cervical conization or simple trachelectomy and pelvic lymphadenectomy was performed after chemotherapy and no recurrences have been reported. Six patients conceived. Maneo et al. [24] report 21 patients with larger tumors <3 cm, in stage IB1, who underwent neoadjuvant chemotherapy (three cycles of isofosfamide, paclitaxel, and cisplatin) followed by conization and pelvic lymphadenectomy. No residual disease was found in five patients and no recurrence is reported after a median follow-up of 69 months. Nine patients have attempted a pregnancy and six became pregnant. There were nine live births with two preterm deliveries and one first trimester miscarriage.

## 6. Conclusions

Recent studies show that there are interesting treatment alternatives to the “golden standard” for patients with early stage of cervical cancer. The available data support the safety of trachelectomy, with a low and acceptable rate of recurrence similar to the traditional options. Vaginal trachelectomy has been the most common approach but abdominal and laparoscopic procedures are now being favored in view of the radicality of excision of parametria. The obstetrical outcome is excellent and the possibility to conceive is high. Prematurity is the main problem. Possible infertility problems due to cervical stenosis can be overcome with dilation or assisted reproductive technologies.

Conization is an option for patients with cervical cancer stage IA1. Recent studies show that conization or simple trachelectomy with pelvic lymphadenectomy could be an alternative in stage IA2 and IB1 with favorable pathologic characteristics, which would reduce the obstetrical risks and perioperative complications. Further research is needed since the studies included a limited number of patients. The role of parametrium thickness and development of imagine technique to measure parametrium volume before surgery is important for the selection of patients who probably benefit from resection of parametrium.

All young patients with diagnosis of cervical cancer should be encouraged to discuss conservative treatment with their gynecologist. Gynecologists should be informed about the fertility sparing options and referred to appropriate gynecologic oncologist. Guidelines and clinical protocols for the management of these patients should be created and followed by the gynecologist involved in the counseling and treatment of these patients.

## Figures and Tables

**Table 1:** Oncological outcomes in patients with early stage cervical cancer who underwent radical vaginal trachelectomy.

	Number of patients	Abandoned trachelectomies	Median followup (months)	Recurrence (*n*)	Deaths (*n*)
Plante et al. 2011 [34]	140	15 (4.8%)	95	6	2
Lanowska et al. 2011 [64]	212	Nr	37	8	4
Pahisa et al. 2008 [14]	15	2	2–95	2	1
Sonoda et al. 2008 [65]	43	2 (4.6%)	21	1	0
Milliken and Shepherd 2008 [66]	158	0	Nr	4	4
Chen et al. 2008 [67]	16	0	28	0	0
Marchiole et al. 2007 [68]	135	17 (4.9%)	95	7	5
Beiner and Covens 2007 [51]	93		30	7	4
Schlaerth et al. 2003 [69]	12	2 (16%)	47	0	0
Burnett et al. 2003 [70]	21	2 (9.5%)	31.5	0	0

Total	845	40	—	35	20

Nr: non reported.

**Table 2:** Tumor characteristics of patients underwent vaginal radical trachelectomy.

	Number of patients	Stage IA1 with LVSI	Stage IA2	Stage IB1	Tumor size <2 cm	Tumor size >2 cm	Median tumor size (cm)	SCC	AC or Adeno SCC	Other histology
Plante et al. 2011 [34]	140	7	30	97	121	19	nr	78	62	—
Lanowska et al. 2011 [64]	212	34	47	131	206	6	nr	154	58	—
Pahisa et al. 2008 [14]	15	0	0	15	12	3	nr	9	6	—
Sonoda et al. 2008 [65]	43	1	7	28	nr	nr	nr	24	19	—
Milliken and Shepherd 2008 [66]	158	—	3	152	nr	nr	nr	103	41	14
Chen et al. 2008 [67]	16	3	7	6	9	7	1.3 (0.2–3)	14	2	—
Marchiole et al. 2007 [68]	118	10	19	83	62	21	1.66 ± 0.91	90	25	3
Beiner and Covens 2007 [51]	93	39	22	31	85	8	nr	40	50	3
Schlaerth et al. 2003 [69]	12	—	8	2	10	2	nr	4	6	2
Burnett et al. 2003 [70]	21	—	1	20	nr	nr	1.1 (0.3–3.0)	12	9	—

LVSI: lymph vascular space involvement, SCC: squamous cell carcinoma, AC: adenocarcinoma, AdenoSCC: adeno squamous cell carcinoma, nr: not reported.

**Table 3:** Oncological outcomes in patients with early stage cervical cancer who underwent abdominal radical trachelectomy.

	Number of patients	Median follow up (months)	Recurrences (*n*)	Deaths (*n*)
Li et al. 2011 [38]	62	22.8	0	0
Muraji et al. 2012 [39]	32	24	0	0
Yao et al. 2010 [71]	10	4–68	0	0
Cibula et al. 2009 [72]	17	21.4	1	0
Nishio et al. 2009 [73]	61	27	6	0
Olawaiye et al. 2009 [74]	10	28	0	0
Pareja et al. 2008 [75]	15	32	0	0
Diaz et al. 2008 [76]	16	12	0	0
Ungár et al. 2005 [77]	30	47	0	0
Rodriguez et al. 2001 [78]	3	9–31	0	0

Total	244	—	7	0

**Table 4:** Tumor characteristics of patients underwent abdominal radical trachelectomy.

	Number of patients	Stage IA1 with LVSI or positive margins at conization	Stage IA′′	Stage IB1	Tumor size (cm) ≤2 cm	Tumor size (cm) >2 cm	Median tumor size (cm)	SCC	AC or Adeno SCC	Other histology
Li et al. 2011 [38]	59	16	7	36	45	14	Nr	50	9	3
Muraji et al. 2012 [39]	23	2	2	19	Nr	Nr	Nr	16r	7	—
Yao et al. 2010 [71]	10	—	5	5	10	—	Nr	8	2	—
Cibula et al. 2009 [72]	24	—	5	5	Nr	Nr	Nr	—	14	10
Nishio et al. 2009 [73]	61	4	8	49	49	12	Nr	58	3	—
Olawaiye et al. 2009 [74]	10	1	3	6	10	—	Nr	3	7	—
Pareja et al. 2008 [75]	15	—	3	12	Nr	Nr	Nr	11	4	—
Diaz et al. 2008 [76]	22	—	—	15	Nr	Nr	1.6	9	13	—
Ungár et al. 2005 [77]	30	—	10	15 (5 stage IB2)	26	4	Nr	26	2	2
Rodriguez et al. 2001 [78]	3	1	2	—	3	—	—	2	1	—

**Table 5:** Oncological outcome in patients with early stage cervical cancer who underwent laparoscopic radical trachelectomy.

	Number of patients	Median followup (months)	Recurrences (*n*)	Deaths (*n*)
Kai-Jiang et al. 2011 [40]	6	8–20	0	0
Wang et al. 2011 [41]	1	14	0	0
Kim et al. 2010 [42]	32	31	1	1
Martin and Torrent 2010 [43]	9	28	1	0
Bafghi et al. 2006 [44]	6	25	1	1
Cibula et al. 2005 [45]	1	9	0	0
Lee et al. 2003 [46]	2	12.9	0	0

Total	57	—	2	2

**Table 6:** Oncological outcome in patients with early stage cervical cancer who underwent robotic radical trachelectomy.

	Number of patients	Median followup (months)	Recurrences (*n*)	Deaths (*n*)
Nick et al. 2012 [49]	12	17 (0.3–64.9)	0	0
Hong et al. 2011 [79]	3	Nr	Nr	Nr
Burnett et al. 2009 [48]	6	9–13	0	0
Chuang et al. 2008 [80]	1	Nr	Nr	Nr
Geisler et al. 2008 [81]	1	Nr	Nr	Nr
Persson et al. 2008 [47]	2	Nr	Nr	Nr

Nr: not reported.

**Table 7:** Reproductive outcome in patients with early cervical cancer who underwent vaginal trachelectomy.

	Number of patients	Total pregnanies	Pregnant women	Firts trimester loss	Pregnant termination /ectopic pregnancies	Second trimester loss	I and II trimester loss	Delivery <32 w	Delivery 32–36 w	Birth after 36 w	Ongoing pregnancies	Total births
Plante et al. 2011 [34]	125	106	58	21	Nr	3	24	4	15	58	Nr	77
Lanowska et al. 2011 [64]	212	60	50	5	2	3	8	3 (<28 w)	15 (28–36 w)	27	4	45
Sonoda et al. 2008 [65]	41	11	11	1	2	0	1	0	0	4	4	4
Pahisa et al. 2010 [14]	13	3	3	0	0	0	0	0	0	1	2	1
Chen et al. 2008 [67]	16	5	5	0	0	2	2	0	1	1	1	2
Shepherd et al. 2008, 2006 [66, 82]	158	88	31	19	3	2	21	10	15	19	7	44
Beiner and Covens 2007 [51]	93	22	18	3	0	3	6	3	3	12	0	18
Schlaerth et al. 2003 [69]	11	4	4	0	0	2	2	0	1	1	0	2
Burnett et al. 2003 [70]	19	4	3	0	0	1	1	0	0	2	1	2
Mathevet et al. 2003 [83]	118	56	34	9	5	8	17	Nr	5 < 36 w	29		34

Total	805	359	217	58	12	24	82	Nm	Nm	154	19	229

Nr: not reported.

Nm: not measurable.
